# Proceedings of the Kansas State Dental Society

**Published:** 1871-11

**Authors:** J. D. Patterson

**Affiliations:** Topeka, Kansas


### PROCEEDINGS OF THE KANSAS STATE DENTAL SOCIETY.

Topeka, Kansas, Sept. 12, 1871.

Society met according to adjournment. President J. B. Wheeler in the chair. Roll was called, and Drs. Fuller, Wheeler, Marvin, Callahan and Patterson answered to their names. Minutes of last meeting were read and adopted. The election of new members was next in order, and the names of A. Doud, of Olathe; L. C. Wasson, of Ottawa; F. De Obert, of Topeka; and C. H. Reeder, of Burlingame, were presented by the membership committee, and after due deliberation they were elected active members of the Society by a unanimous vote, signed the Constitution, and paid the required fee. Society then adjourned to meet at 9 o'clock to-morrow at the parlors of the Fifth Avenue Hotel.

Sept. 13th, 9 o'clock. Meeting called to order by the president, all of the members present. Discussion on operative dentistry ensued.

Dr. Fuller said, I have been filling teeth for the last two years with foil; I use the hand mallet of lignum vitæ; have an assistant all the time. I have an automatic plugger, but use it very little, and prefer the hand mallet used by an assistant.

L. C. Wasson. I strongly advocate the use of Watts'

crystal gold; have been using it for the last ten years; commenced its use when first introduced, have used it with great success, and am quite sure that I can make a better filling with it than with foil. I know that a great many differ with me, and that the profession generally is opposed to its use. I think I can restore the lost part of a tooth better and more rapidly, and with the same amount of labor as with foil. Have not replaced more than two fillings where I used Watts' crystal gold. I owe my success with crystal gold to my instructions in using it by good operators and advocates of this form of gold; think the trouble is with many operators that they are not careful enough in its use. I use instruments with very shallow serrations. I have not a word to say against foil.

E. C. Fuller. I think if crystal gold is carefully worked with shallow serrated instruments, a good filling can be made with it. I confess that I do not know how to work it. I use heavy foil especially for building out lost portions of a tooth. I can work it nearly as fast as amalgam. I do not use No. 120 as much as formerly, but smaller numbers of the heavy foil. Think it is not necessary to say much about amalgam. I use os-artificiel for capping nerves—leave it a few days in sensitive cavities to remove sensitiveness. Over exposed nerves I fill with os artificiel, afterward remove and fill with gold.

J. B. Wheeler. Would it not be better to leave some of the os-artificiel?

E. C. Fuller. Yes, I always endeavor to do so, but sometimes find it very difficult. I think that new dentine is often formed.

L. C. Wasson. I have been using os-artificiel for some time with flattering results. I have seen a good deal about the formation of new dentine, especially in the reports of the Illinois Society. I do not think new dentine forms after the patient is twenty-five years old. I had a case of exposed nerve filled with Hill's stopping; after six months removed and found new dentine formed. Have not had good success in capping nerves in the teeth of old persons.

E. C. Fuller. I do not think best to remove the os-artificiel in so short a time; think it ought to remain for a year if circumstances do not compel the hastening of filling with gold.

A. M. Callahan. I succeed very well in capping with young persons, not with old; with elderly people I destroy an exposed nerve, and make a fang filling. Sometimes fail with young persons also. Many circumstances and conditions regarding the patient influence the success of the operation of capping nerves.

A. Doud. I have not had much experience with os-artificiel. Have used it some. In one case of nerve exposure I filled with os artificiel; nerve was healthy—was compelled to remove the filling on account of pain. My experience is that the use of os-artificiel is almost always accompanied with pain, often very severe, and sometimes destroys an exposed nerve.

L. C. Wasson. The pain is generally saved by cauterization; am careful not to put the os-artificiel in when in too fluid a state, else it will produce more pain than when thick and pasty.

E. C. Fuller. It requires considerable skill to insert an os-artificiel filling; I introduce it so that it does not require pressing.

L. C. Wasson. I do not agree with Dr. Fuller as to the proper consistency of os-artificiel when being introduced. Do not think the fluid portion of the preparation amounts to anything.

Dr. Hewett, of Kansas City, Mo., was in attendance, and was elected an honorary member.

Dr. Hewett said. I have good success with os-artificiel, and use it in the same manner as Dr. Wasson does, and leave it in as long as it will last. I use the Eureka gold filling; it is much tougher than foil, and I prefer it to any other form of gold; have good success in building out with it; use shallow serrated instruments.

C. H. Reeder. I have watched closely the interest in os-artificiel. I use collodion on an exposed nerve before filling with os-artificiel, and recommend it highly. I am using M. M. Johnson's foil.

Dr. Marvin. I coincide with Dr. Wasson in regard to the use of sponge gold.

Dr. Wheeler. Have been and am using os-artificiel. I have a case of three molars with exposed nerve, os-artificiel filling have been in for nearly a year, I intend replacing with gold in a short time, leaving part of the zinc. A question with me is, whether decay will be more likely to ensue with os-artificiel over the nerve than with gold.

E. C. Fuller. I think, when possible, it is probably better to remove all of the zinc. I saw a case filled by Morrison, of St. Louis, where the os-artificiel apparently softened the dentine.

Dr. Wheeler. I think such is often the case. In filling, I use the rubber dam to a limited extent; the more I use it the better I like it. I made a failure with crystal gold, however; it may be the fault was mine. I have injured my practice with it. Do not like Morgan's gold.

Dr. Marvin. I used Lamm's gold, but became disgusted with it.

Dr. Wheeler. I think that men in high standing in the profession did wrong in giving favorable certificates to Lamm's and Morgan's gold.

Dr. DeObert. Can os-artificiel always be used on exposed nerves? is it advisable to use it after considerable pain has ensued by exposure?

Dr. Wheeler. Not always. I would ask Dr. Wasson if he uses Morgan's gold?

Dr. Wasson. I have never used either Morgan's or Lamm's, but am much pleased with the Eureka gold.

Dr. Wheeler. I always cap a healthy nerve, whether in old or young persons. I think it often dies in old persons, but often the foramen is so closed that no harmful result ensues from the putrid matter engendering inflammation. A lady applied to me to have a tooth pivoted, the root healthy; some would have preferred building out, but I decided to pivot; there was no sign of a pulp chamber; but after drilling nearly to the end of the root, I found a healthy nerve I often fail with ulcerated teeth and cases of abscess. I think the hygienic treatment is not used enough; have used creosote, iodine, etc.; better let medicine alone, and cleanse better. I prefer in superior teeth to not stop the cavity at all; give plenty of time. I would not try to cure when scurvy is in the system.

Dr. Callahan. How do you get rid of the pus in the lower jaw.

Dr. Wheeler. It is difficult. I cleanse often, and keep the cavity closed.

Dr. Patterson. I have paid considerable attention to this class of cases, and in practice have often been successful enough so as to warrant zealous perseverance to cure ulcerated teeth. Have not used the heroic treatment, but rather the palliative. In upper cases I do not close the cavity, so that matter can not escape from the root; a pellet of loose cotton in the cavity of decay will absorb any discharge, and prevent unpleasantness in the mouth. I am very careful not to be in haste about filling. I have found cases that I could not cure so as to make it safe to fill in the usual manner, and I adopted a mode of procedure given in the *American Journal of Dental Science* by J. L. Dodge; cleaned out the cavity and roots thoroughly, filled the crown cavity and major part of the pulp cavity, leaving the nerve cavity and lower part of the pulp chamber vacant; and after completing, made an opening with a small drill under the edge of the gum into the unfilled portion of the pulp cavity, affording a passage for any matter that would accumulate. I have four of these cases, some of one year's standing, and the teeth have not given trouble. The discharge, if any, is not noticed. I have seen but two cases where the heroic treatment of cutting the secreting sac from the root with an instrument was used, and the treatment in both cases failed; one of them was in my own mouth.

Dr. Wasson. In a case of ulceration of an upper molar, I cleansed with tepid water; could not reduce the inflammation; I then introduced a tent through the fistula, treated with carbolic acid and iodine, and finally cured and filled with gold; it is now solid.

Society now adjourned to meet at 2 o'clock at Dr. Callahan's office for the purpose of witnessing a clinic by Dr. Wheeler, viz., the filling of a tooth, using the rubber dam, when a difficult case was successfully filled.

In the evening the Society met and discussed mechanical dentistry.

Dr. Callahan. I am using rubber almost entirely as a base. I put up a few cases of Watt and Williams' metal, and one of Weston's metal. I prefer Watts' base to that of Weston's.

Dr. Reeder. Have used rubber with very good success. Some claim that rubber can not be worn without detriment to the mouth. I do not think there is cause for such fear.

Dr. Marvin. I now never take an impression in wax, and get along very much better. Do not use metallic plates for upper sets, sometimes I do for lower. I sometimes load a lower rubber plate with lead; weight is very necessary in lower plates, make air chambers with lead. Some plates should have a deeper chamber than others.

Dr. Doud. Our processes for rubber are much the same. I use tin for air chambers, and coat my cast with liquid silex: take impressions for partial cases with wax.

Dr. Patterson. My process for rubber is much the same as that of those who have spoken. I think there is not enough attention paid to this department of dentistry. I think there is, in inserting artificial teeth, room for the exercise of a higher talent, than inserting fillings afford, only to a limited extent, however, when block gum teeth are used. I use the plain teeth whenever I can. Operative dentistry of course merits our first attention, as its aim is the preservation of the natural teeth; but the replacing of the natural organs with artificial ones has not received the attention it deserves, and for that reason has been a fruitful source of charlatanism, and I think it is the fault of the profession that it is so. As long as we give so little attention to this department of dentistry, we must not find fault with "cheap work," because we *treat* it as cheap work; if the insertion of artificial teeth requires no skill save purely mechanical,—no dental education as many claim,—then let us give it over entirely to mechanics and those who have no dental education.

E. C. Fuller. As far as I have used rubber, it certainly has merits above the other bases. I feel biased in favor of operative dentistry as compared with mechanical, and am an advocate of the use of plain teeth—can articulate better with them. Do not believe in loading the rubber with lead. I like Weston's metal tolerably well; have never used Watt and Williams'.

L. C. Wasson. I use soap to separate. I do not think air chambers are necessary. I think operative dentistry is superior to mechanical dentistry; in making artificial teeth the process is the same in every case and purely mechanical.

Dr. Wheeler. I have seen dangerous effects of red rubber. I think in many cases it is wrong to put it in. In a case where a patient wore red rubber, he complained of an itching sensation in the parts contiguous to the plate. I made a plate of black rubber, and the difficulty was entirely avoided.

E. C. Fuller. Have noticed ill effects from wearing a plate. Do not know whether it was effect from mercury or not.

L C. Wasson. I think a plate of any material will often produce ill effects; do not attribute it to red rubber.

The discussion here turned to filling teeth with amalgam, and the practice was condemned; tin was recommended to be used for cheap filling.

The general interests of the Society were discussed at length, then adjourned to meet at Atchison, May 2d, 1872.

J. D. PATTERSON, *Recording Sec'y.*

